# The *Children and Parents in Focus* project: a population-based cluster-randomised controlled trial to prevent behavioural and emotional problems in children

**DOI:** 10.1186/1471-2458-13-961

**Published:** 2013-10-16

**Authors:** Raziye Salari, Helena Fabian, Ron Prinz, Steven Lucas, Inna Feldman, Amanda Fairchild, Anna Sarkadi

**Affiliations:** 1 Department of Women’s and Children’s Health, Samariterhemmets Hospital, Uppsala University, Box 609, 751 25 Uppsala, Sweden; 2 Parenting and Family Research Center, University of South Carolina, Columbia, USA

**Keywords:** Universal and selective prevention, Parenting programmes, Preschool children, Externalising problems

## Abstract

**Background:**

There is large body of knowledge to support the importance of early interventions to improve child health and development. Nonetheless, it is important to identify cost-effective blends of preventive interventions with adequate coverage and feasible delivery modes. The aim of the Children and Parents in Focus trial is to compare two levels of parenting programme intensity and rate of exposure, with a control condition to address impact and cost-effectiveness of a universally offered evidence-based parenting programme in the Swedish context.

**Methods/Design:**

The trial has a cluster randomised controlled design comprising three arms: Universal arm (with access to participation in *Triple P - Positive Parenting Program*, level 2); Universal Plus arm (with access to participation in *Triple P - Positive Parenting Program*, level 2 as well as level 3, and level 4 group); and Services as Usual arm. The sampling frame is Uppsala municipality in Sweden. Child health centres consecutively recruit parents of children aged 3 to 5 years before their yearly check-ups (during the years 2013–2017). Outcomes will be measured annually. The primary outcome will be children’s behavioural and emotional problems as rated by three informants: fathers, mothers and preschool teachers. The other outcomes will be parents’ behaviour and parents’ general health. Health economic evaluations will analyse cost-effectiveness of the interventions versus care as usual by comparing the costs and consequences in terms of impact on children’s mental health, parent’s mental health and health-related quality of life.

**Discussion:**

This study addresses the need for comprehensive evaluation of the long-term effects, costs and benefits of early parenting interventions embedded within existing systems. In addition, the study will generate population-based data on the mental health and well-being of preschool aged children in Sweden.

**Trial registration:**

ISRCTN: ISRCTN16513449.

## Introduction

Parenting support programmes have proven to be effective in children with different levels of externalising behaviour problems [1]. Efforts to offer these programmes more universally have reported small effect sizes with low or no significance levels [2,3]. Therefore, the question arises as to whether it is a matter of programme content, mode of delivery, level of intensity, rate of exposure, all, or a combination of the above that gives rise to conflicting evidence on the benefit of parenting programmes offered to non-selected groups. The aim of the Children and Parents in Focus project is to compare two levels of programme intensity and rate of exposure with a control condition to address impact and cost-effectiveness of a universally offered evidence-based parenting programme in the Swedish context.

## Background

Sweden is one of the countries with the best child health statistics in the world. Sweden’s parental leave policies are a subject of envy for many countries [4]. The overall physical health of Swedish children is also excellent. However, in an overview of Swedish research, the Royal Academy of Science in Sweden noted that there is almost no data on the mental health and well-being of preschool aged children [5]. This is especially unfortunate as there is a large body of knowledge to support the importance of early interventions to improve child health and development.

There is broad consensus that prevention and early intervention are more effective than treating problems after they have developed. To decrease the burden of mental health problems in children, one can choose different approaches. Universal programmes might be seen as either too costly or too broad, while selective programmes are potentially stigmatising and ineffective in reaching their target population [6,7]. To reduce prevalence rates of mental health problems among children, it is important to identify cost-effective blends of preventive interventions with adequate coverage and feasible delivery modes [8] as well as effective barrier reducing strategies [9,10].

Exactly what blend of universal, targeted and indicated approaches should be used depends on the prevalence of the condition, the efficacy and cost of intervention at different problem levels and the potential to screen for the condition [11]. For most conditions, different blends of universal, targeted and indicated approaches are needed. Figuring out what that blend should exactly look like is, however, still an empirical question. For ubiquitous conditions, such as behavioural problems in children, the universal approach is motivated given the high societal costs of disruptive behaviour during adolescence, the low cost of early interventions and the insecurity of screening instruments’ predictive value over time. Nevertheless, should a universal approach be combined with a targeted or indicated approach?

Currently, there is an ongoing population-based cluster-randomised controlled trial in Australia investigating how behaviour problems in children can be prevented by using a three-arm design [12]. One arm is a universal approach completed by an indicated approach, whereas the other arm is an indicated only arm where families at risk are offered an intervention and the third arm is care as usual. Our study offers a potentially useful complement to the Australian study in that we compare a universal only with a universal plus indicated approach and care as usual. Compiling the findings from these two studies together might provide important answers as to which approach has the best uptake and is most cost-effective.

### The Swedish scene for preventive services

The Swedish Child Health Centres (CHCs) provide a free service that reaches 99% of the 0–5-year-old population [13]. The centres are publicly funded facilities that may be either publicly or privately run. Part of the success of the CHCs is the relationship that is developed between the CHC nurse and the family during the infant’s first year, during which time a CHC nurse typically sees the infant 11 to 13 times [14]. After infancy, children in Sweden typically see the CHC nurse for an additional 4 to 9 times before age six, provided there are no serious health problems.

The Swedish CHCs have a history that goes back to the 1930s [15]. Over the decades, CHCs have evolved from being a medical, disease-centred model to a more social-based model with greater emphasis on health promotion, empowerment and social support to new parents [16]. However, the core of the CHC programme is still that of screening for developmental delay and serious disease, preventing communicable diseases through vaccination, as well as accident and injury prevention. In 1999, a consensus conference [17] underlined the need to update the screening and interventions offered by the CHCs, as much of what was being done lacked a sufficient evidence-base. Despite some improvement in services, the only screening with a clear evidence-base is the language screening employed at three years of age [18] for the 3–5-years-old age group. Therefore, there is a clear need to improve the content of CHC services for this age group, both in terms of the screening tools used and interventions offered.

Sweden has a preschool system that is of high quality as well as affordable to all families with preschool-aged children. The system is publicly financed through taxes, but the individual preschools can be both publicly and privately run. The same fees apply in both settings; the difference is the organisational form as the public preschools are run by the municipalities, whereas the private preschools are owned by companies, foundations or cooperatives of parents or teachers. The attendance rate is high and 83% of all 1–5-year-olds attend preschool [19]. Therefore, preschools seem to be an ideal arena to reach parents of preschool children. In a previous study when a parenting programme was offered at the local preschools, parents welcomed the model and felt that the greatest advantage was the low threshold to receive the service and that they did not feel stigmatised when attending the sessions offered [20].

### Aims

The aim of this paper is to provide a detailed study protocol of a controlled preschool randomisation trial, including 7,600 children. The aim of the Children and Parents in Focus trial is to compare two levels of programme intensity and rate of exposure, with a control condition to address impact and cost-effectiveness of a universally offered evidence-based parenting programme in the Swedish context of preventive services.

## Methods/Design

### Design

The trial is a cluster-randomised controlled trial with three arms: Universal arm (with a universal offer to participate in *Triple P - Positive Parenting Program*, level 2); Universal Plus arm (with offer to participate in *Triple P - Positive Parenting Program*, level 2 as well as level 3, and level 4 group); and Services as Usual arm. The trial runs from 2013 to 2017.

### Funding and ethics approval

The trial is funded by a joint grant from FORMAS, Vetenskapsrådet, FAS and VINNOVA (grant number 259-2012-68) and approved by the Regional Ethical Review Board in Uppsala (document number 2012/437). The trial is also registered with an international trial registry (ISRCTN16513449).

### Recruitment process

The sampling frame consists of Uppsala municipality, Sweden, (population 202,625). Child health centres (CHC) will consecutively recruit parents of children aged 3 to 5 years before their yearly check-ups (during the years 2013–2017). This implies that each year there will be a completely new cohort of three-year-olds entering the study. Cohorts of four and five-year-olds might also change slightly as some families move in and out of the municipality. In May 2013, the total number of children aged three to five who resided in the catchment area was N = 7,638. Of these n = 2,603 were born 2010; n = 2,494 were born 2009 and 2,541 in 2008. Part of the sample is longitudinal, i.e. children who are three to four years of age at the onset of the study will be followed until they are five-years-old.

#### Child Health Centres (CHC)

All (n = 28) CHCs serving Uppsala municipality were contacted after the approval from the ethics committee and the head of primary health care. Informational meetings were held with the CHC nurses and all heads of the primary health care clinics were invited to participate through passive consent. All the invited CHCs agreed to participate.

#### Parents

As part of the routine care in Sweden, about three weeks before the child’s birthday, nurses at CHCs send out a letter to invite the child’s legal custodians to their yearly developmental check-up. Attached to the letter, there are a few forms that need to be completed by only one of the child’s legal custodians. The accompanying parent is instructed to bring these forms along to the visit.

This standard procedure will change slightly in CHCs that participate in the study. Nurses in these CHCs will attach a new package to the invitation letter. This package will include two sets of the following forms (one for each of the child’s legal custodians when applicable): study information sheets, consent forms and study questionnaires. The package will also contain an additional questionnaire to be completed by the child’s preschool teacher. If the parents decide to participate in the study, they will be asked to take this questionnaire to the child’s preschool and leave it with the child’s teacher. Teachers are then instructed to complete the questionnaire and send it back to the child’s respective CHC in a prepaid envelope. Parents are asked to take their signed consent form and their completed questionnaires with them when attending their child’s check-up at the CHC.

#### Preschools

There are 13 preschool administrative areas in Uppsala municipality. The preschool leadership in the municipality accepted to participate in the project. It was agreed that the administrative areas would be randomised into the three arms of the study with all preschools within an area automatically belonging to the allocated arm of that area.

### Inclusion criteria for the study

All parents of 3, 4 and 5-year-old children who attend the check-up can participate in the study.

### Inclusion criteria for the intervention

All parents of 3, 4 and 5-year-old children who attend the intervention preschools can attend the intervention.

### Exclusion criteria for the study

A translation of the study information and questionnaire is provided in the five most common languages in the immigrant community in Uppsala: Arabic, Somali, Persian, Sorani and English. Parents will be excluded from the study if they neither understand Swedish nor any of the above languages.

### Exclusion criteria for the intervention

Intervention is available in Swedish and Arabic, but not in any other languages. Although interpreters will sometimes be made available, lack of interpreter or language skills function as exclusion criteria.

### Allocation

Allocation was performed through randomising the 13 areas into the three arms of the study. The randomisation was conducted by the preschool leadership at one of their team meetings. Names of all the areas were written on pieces of papers and put in a container. Thereafter, consecutive allocation to groups 1, 2 and 3 took place as the pieces of paper were drawn from the container, where group 1 was the Universal Plus arm, group 2 the Universal arm and group 3 the Services as Usual arm. Four persons witnessed the randomisation process and no changes in allocation were made after the draw. Five areas were allocated to the Universal Plus arm, four to the Universal arm and four to the Services as Usual arm. Individual preschools in each area automatically belonged to the allocated arm for that area.

Because the trained practitioners will not necessarily have to work at the allocated preschools, the intervention preschools will not need to send their own personnel for training, but will be served by practitioners from other preschools. Similarly, preschool teachers with previous Triple P training who work at preschools in the control areas will not be allowed to deliver Triple P in their own preschools. The training costs will be covered by the research grant, whereas implementation costs will be covered by the municipality.

### Intervention

Triple P is a multilevel system of parenting and family support interventions [21], based on social learning theory [22], which aims to reduce behavioural and emotional problems in children by increasing parents’ knowledge, skills and confidence. It consists of five levels of intervention with increasing intensity. Level 1 is a universal intervention targeted towards all parents and provides general information about parenting. Level 5 is an enhanced intervention for parents of children with behavioural problems who also experience other difficulties, such as parental depression or marital conflict. Numerous studies have shown that Triple P is effective in improving parenting behaviour for a review see, [23] and reducing children’s problem behaviours for a review see, [24]. In this study, Triple P level 2 will be offered as a universal programme in all the preschools in both Universal and Universal Plus arms, while Triple P level 3 and 4 will only be offered at preschools in the Universal Plus arm. The term universal here is meant to convey that all parents have access to the programme. The goal is not to have 100% of parents participate in the programme.

Level 2, Selected Triple P, is a low intensity parenting intervention targeted towards parents who seek general information about parenting or have specific concerns about their child’s development or behaviour. It can be delivered as large group seminars or brief individual consultations. The large group seminars, called the Triple P Seminar Series, involve three stand-alone parenting seminars: The Power of Positive Parenting; Raising Confident, Competent Children; and Raising Resilient Children. Each seminar lasts about 90 minutes and includes a presentation as well as a question-and-answer section. Parents receive a parenting tips sheet and can approach the facilitator for individual inquiries. The brief consultations involve one or two consultation contacts with individual parents and each contact lasting up to 20 minutes. Active skill training is not included in this level of Triple P.

Level 3, Primary Care Triple P, is a low to moderate intensity parenting intervention targeted towards parents who have specific and discrete concerns about their child’s behaviour or development and need individual assistance. It consists of up to four brief (15 to 30 minutes) individual consultations and incorporates a combination of advice and information as well as active skills training strategies such as, rehearsal and self-evaluation.

Level 4, Group Triple P, is a moderate to high intensity parenting intervention targeted towards parents who show clear deficits in their parenting skills and have children with multiple problems in a variety of settings. It consists of five two-hour group sessions and three 15 to 30 minute individual telephone consultations. During the group sessions, parents learn a range of child management skills through discussion, modelling, rehearsal, feedback and between sessions practice. During the telephone sessions, parents receive additional support while practicing the skills that they have learnt in the group.

### Outcome measures

Outcomes will be measured annually. The primary outcome for children will be measured by Strength and Difficulties Questionnaires (SDQ) [25,26] collected from three sources: fathers, mothers and preschool teachers. The SDQ measures both prosocial and difficult behaviours in children aged 3 to 16. It consists of 25 items on a 3-point scale from 0 (not true) to 2 (certainly true). The 25 items form five subscales: Hyperactivity, Emotional Symptoms, Conduct Problems, Peer Problems and Pro-social. A Total Difficulties score is computed by summing up the scores for the first four subscales. The extended version assesses whether the respondent thinks the child has a problem, and if so, the perceived impact on the child and the burden on the family. The SDQ has good internal consistency, test-retest reliability and discriminant validity [27,28].

Parents’ behaviour will be measured using the Parenting Scale (PS) [29]. When applicable, we will ask both mothers and fathers to complete the PS. The PS is a 30-item questionnaire using a 7-point scale, which measures the parents’ dysfunctional discipline styles by asking them to rate how they would respond to a specific behavioural problem. The scale has adequate to good internal consistency and test-retest reliability [29]. We will use the Swedish version of the scale [30], which retains only two of the three original subscales: Laxness (11 items), which is associated with permissive and inconsistent parenting, and Overreactivity (10 items), which is characterised by harsh and punitive discipline.

Parents’ general health will be measured by General Health Questionnaire (GHQ-12) [31], which has 12 items, each assessing the severity of a mental problem over the past few weeks using a 4-point scale (from 0 to 3). The instrument is well validated among adults and widely used in many countries.

Table 1 lists all the background information and constructs measured in the study. Parents and teachers complete a single assessment package for each child once every year. The practitioners complete their forms as part of each intervention session. These forms will first be used to specify individual exposure and then be aggregated yearly to form the numbers for each specific year.

**Table 1:** Background and outcome measures in the study

		**Purpose**		
**Construct**	**Measure**	**CHC use**	**Effects measure**	**Health economy**
**Parent questionnaire measures**				
Child basic information	- Date of birth	**χ**	**χ**	**χ**
	- Sex			
Child general health	- General health	**χ**		**χ**
	- Disability			
	- Chronic illness			
	- Visits to physicians			
	- Admission to hospital			
	- Asthma and common allergies			
Language problems	- Receptive language	**χ**		
	- Expressive language (only for 4- and 5-year-olds)			
Autism spectrum symptoms	- Role play	**χ**		
	- Short conversations			
	- Changing routines			
Motor skills (only for 5-year-olds)	- Gross motor skills (e.g. running)	**χ**		
	- Fine motor skills (e.g. drawing)			
Child behavioural and emotional problems	- The strengths and difficulties questionnaire (extended version)	**χ**	**χ**	**χ**
Service use (at home or preschool)	- Special education			**χ**
	- Counsellor			
	- Psychologist			
	- Assistant in classroom			
	- Physiotherapist			
	- Speech therapist			
	- Social worker			
	- Parenting programmes			
Parent health	- General health questionnaire		**χ**	**χ**
	- Self-rated health question			
Parenting behaviour	- The parenting scale		**χ**	
Language stimulation	- Talking to child		**χ**	
	- Reading to child			
Background information for parent	- Age		**χ**	**χ**
	- Relationship status			
	- Education			
	- Job status			
	- Number and age of children			
	- Country of birth			
	- Intention to move			
	- Type of household			
Parent engagement	- Accessibility		**χ**	
	- Engagement			
	- Responsibility			
**Teacher questionnaire measures**				
Child behavioural and emotional problems	- The strengths and difficulties questionnaire	**χ**	**χ**	**χ**
Language problems	- Receptive language	**χ**		
	- Expressive language (only for 4- and 5-year-olds)			
Autism spectrum symptoms	- Role play	**χ**		
	- Short conversations			
	- Changing routines			
Motor skills (only for 5-year-olds)	- Gross motor skills (e.g. running)	**χ**		
	- Fine motor skills (e.g. drawing)			
**Practitioners forms**				
Intervention fidelity	- Fidelity checklist		**χ**	
Intervention delivery	- Number of sessions held per level		**χ**	**χ**
	- Number of participants in each session			
Intervention exposure	- List of participating parents		**χ**	**χ**

### Health economic evaluation

To estimate health outcomes in *Quality Adjusted Life Years* (QALY), GHQ12 will be transformed into quality weights (EQ-5D index) [32] that represent the health-related quality of life of the health state under consideration. This will be done using cross-reference between GHQ-12 and EQ-5D index in population survey data available in Uppsala county that includes both GHQ-12 and EQ-5D [33].

Costs of delivering each intervention will be measured using a societal perspective including both *direct costs*, such as training, material and premises, and *indirect costs*, such as participant leisure time and productivity losses. Costs of families’ use of health and social services outside of the study, such as contacts with social services and health care, psychologist, counsellor, school assistant and special educator will be measured by parental report. Unit costs for measured resource use will be estimated using official statistics databases.

### Procedure

#### First year (August 2013 – August 2014)

During the first year of the project, the procedure will be the same for all the three arms. CHCs in the study provide their usual health and developmental check-up of preschool children with an addition of the study questionnaires.

A manual is developed for nurses to follow when they send out the questionnaires to parents, including uniform instructions on how to collect and interpret questions about the child’s health and behaviour during the visit. The manual includes transparent sheets to overlay the questionnaires in order to highlight whether the child has problems in certain areas. Each of the following areas is highlighted in its own colours: physical health, language skills, peer problems and social skills, behaviour and emotional problems and motor development. Some questions are marked with specific instructions about where to refer the child if further help is indicated. In the transparent overlay for the SDQ, the 25 response alternatives are marked with red or yellow colours, corresponding to the problematic behaviour (scored as 2 in the four difficulties subscales and 0 in the Prosocial subscale). The red colour indicates behavioural and emotional problems (Conduct Problems, Hyperactivity and Emotional Symptoms), while the yellow colour indicates social problems (Peer Problems and Prosocial subscales).

Nurses will be informed that a child is most likely to have problems if: the child’s scores mainly fall in the red or yellow areas or if parents are concerned about their child’s behaviour and/or the teacher indicates that the child has definite or severe difficulties (impact question). Nurses will not be instructed to score the SDQ or to refer children based on their SDQ scores.

#### Following years (August 2014 – May 2017)

In the consecutive years, while procedure for data collection (at CHCs) remains unchanged across the three arms, preschools in the Universal and Universal Plus arms will start the dissemination of the interventions. Preschools in the Universal arm will only offer Triple P Seminar Series plus the short individual counselling within level 2. Preschools in the Universal Plus arm will offer Triple P Seminars as well as Triple P Primary Care and Triple P Group.

All three levels of intervention will be advertised for parents of 3- to 5-year-old children, but they will be open to all parents. Targeted parents are free to accept or decline the offer. We expect about 30 to 40% of the families in both intervention arms to attend at least one of the seminar sessions. We expect the uptake of the Primary Care and Group Triple P to be between 10 to 20% in the Universal Plus arm.

### Analytic plan

#### Population level analysis

A key question in the study pertains to evaluating the impact of the intervention arms at the level of the preschool, i.e. at a population level. This analysis will examine how the intervention conditions impact aggregate children’s social-emotional and behavioural health as reported by parents and preschool teachers. Mean level scores are of interest, as well as proportion of children above the clinical cut-off on the instrument. The baseline year will include all children ages 3–5 in the preschools. The second year will again include all children 3–5 years of age in the preschools, with only the continuing 4-year-olds and 5-year-olds having been assessed in the baseline year. Similarly, the third year, which again will include all children 3–5 years of age in the preschools, but with only the continuing 5-year-olds having been assessed in the two prior years. Focusing only on aggregate effects at the level of the preschool, ANCOVA will be conducted with the three arms, controlling for baseline and other preschool-level covariates (e.g. socioeconomic level).

#### Longitudinal analysis

The design also permits examination of how individual children (nested within preschools) progress longitudinally with respect to outcome measures, as a function to intervention exposure. Specifically, children who are age 3 years during the baseline year will be followed longitudinally through the second and third years (i.e. from age 3 to age 5). Similarly, the children who are age 4 years during the baseline year will be followed longitudinally through the second year. Hierarchical linear modelling, which will account for clustering, will be used to test the effects of intervention conditions, controlling for baseline and other covariates. Assuming an alpha of .05 and 80% power, it is estimated that the design as planned permits detection of effect sizes ranging from .17 for an intraclass correlation of 0.02 to .26 for an intraclass correlation of 0.10. The study is powered to detect small to moderately small effects.

#### Cost-effectiveness analysis

Economic evaluation will be presented first as a cost-consequences analysis [34], which allows policy makers to compare the incremental costs with all outcomes of interest – i.e. child behaviour, parent’s mental health, etc. and impact on parent’s health-related quality of life. Economic evaluation will then present cost-effectiveness analyses, comparing incremental costs to incremental changes in child behaviour and a cost-utility analysis, comparing incremental costs to incremental parental quality of life (as measured by GHQ12 transformed to EQ-5D). The results of the analysis will be expressed as *incremental cost-effectiveness ratios* (ICER) [35] where differences in costs are compared to differences in health consequences (Δcost/ΔQALY) for each intervention.

A decision analytical model will be developed to perform long-term prognostic calculations of potential health effects and costs savings due to reduction of child mental health problems. Analyses of long-term cost-effectiveness of early intervention will be provided.

## Discussion

The preschool years constitute one of the most sensitive periods in a child’s development and several early intervention strategies are available. There is a paucity of research on the long-term effects of structured parenting programmes offered to the population at large. Sweden has a tradition of universal parenting education through individual contact, but the effects of specific interventions are seldom evaluated within the system. Health economic evaluations are imperative for decision makers, but most parenting interventions lack such data. If Sweden is to keep using its resources in an equitable and cost-effective way, while keeping its international forefront position, it is time to empirically develop an effective blend of culturally appropriate interventions to change the prevalence of child mental health problems.

This study addresses the need for comprehensive evaluation of the long-term effects, costs and benefits of early parenting interventions embedded within existing systems. In addition, the study will generate population-based data on the mental health and well-being of preschool aged children in Sweden as currently, there is almost no such data available [5].

## Abbreviations

CHC: Child health centres; Triple P: Positive Parenting Program; SDQ: Strength and Difficulties Questionnaires; PS: Parenting Scale; GHQ-12: General Health Questionnaire; QALY: Quality Adjusted Life Years; ICER: Incremental cost-effectiveness ratios.

## Competing interests

RS is a contract trainer and RP is a consultant to Triple P International, which disseminates the Triple P programme. The other authors declare that they have no competing interest.

## Authors’ contributions

AS is the principal investigator. All authors have been involved in the conception and design of the study. RP and AF provided statistical expertise in the trial design and RS is conducting the primary statistical analysis. IF developed the health economic evaluation. AS, HF and SL helped with the implementation. All authors contributed to refinement of the study protocol and approved the final manuscript.

## Pre-publication history

The pre-publication history for this paper can be accessed here:

http://www.biomedcentral.com/1471-2458/13/961/prepub
